# Supplementary material for: Influence of an oral health promotion program on the evolution of dental status in New Caledonia: A focus on health inequities
Source: PLoS One. 2023 Oct 3;18(10):e0287067. doi: 10.1371/journal.pone.0287067 (PMC10547163; doi:10.1371/journal.pone.0287067)
Supplement: S1 Table — (DOCX) [file pone.0287067.s001.docx]

S1 Table: Description of the study explanatory variables **(2019)**

| Variables | Answer options | Source data | | | |
| --- | --- | --- | --- | --- | --- |
|  |  | School register | Child questionnaire | Parent questionnaire | Clinical examination |
| **Sociodemographic status, Ethnicity & conditions of living** | |  |  |  |  |
| Gender | Male**;** Female |  |  |  |  |
| province | South**;** North**;** Islands |  |  |  |  |
| Ethnicity | Oceanian**;** European**;** Multiracial**;** Other |  |  |  |  |
| Place of living | Tribe/squat**;** Town/village/Countryside |  |  |  |  |
| Health insurance * | Basic public insurance only**;** State aid supplemental**;** Private supplemental |  |  |  |  |
| Type of school | Public; Private |  |  |  |  |
| Sanitary equipment  (bathroom/shower/hot water/toilet) | All available; one or more missing |  |  |  |  |
| **Access to oral health care and prevention** | |  |  |  |  |
| Number of sealed molars + | None;1 to 3; =4 |  |  |  |  |
| Tooth brushing at school | Yes; No |  |  |  |  |
| Dental attendance | Never visited a dentist; Already had; Visit to the dentist every year |  |  |  |  |
| Access to oral health care | No difficulties; Difficulties^&^ |  |  |  |  |
| Participation to the OHP program | Yes; No |  |  |  |  |
| **Oral health behaviours** | |  |  |  |  |
| Frequency of tooth brushing | Twice a day or more; Once a day or less |  |  |  |  |
| Usual drink when thirsty | Sweet drink/Milk; water |  |  |  |  |
| Usual drink during mealtime | Sweet drink/Milk; water |  |  |  |  |
| Sweet drinks during weekdays | Never, Sometimes, Daily |  |  |  |  |
| Sweet foods during weekdays | Never, Sometimes, Daily |  |  |  |  |
| Breakfast before school | Never, Sometimes, Daily |  |  |  |  |

grey area: source of information

+ no sign of dental sealant material vs partial (some part of the occlusal fissures with dental sealant material) or complete (all occlusal fissures with sealant material) presence of dental sealant

*Basic public health insurance in NC is covering a large proportion of essential oral health care fees. Supplementary state aid (for low income earners) and private insurances complete this dental coverage and potentially give extra reimbursements for other types of dental treatments such as prosthetic treatments. Dental care is deleivered in private practices or public structures.

^&^Parents answered the question : During the last year, did you attempt to attend the dentist but could not get an access to oral health care for your child?
