# Supplementary material for: Influence of an oral health promotion program on the evolution of dental status in New Caledonia: A focus on health inequities
Source: PLoS One. 2023 Oct 3;18(10):e0287067. doi: 10.1371/journal.pone.0287067 (PMC10547163; doi:10.1371/journal.pone.0287067)
Supplement: S2 Table — (DOCX) [file pone.0287067.s002.docx]

S2 Table: Description of the study population for the explanatory variables by ethnicity **(2019)**

| Variables | Oceanian | | European/ Others | | Multiracial | | Total | | p |
| --- | --- | --- | --- | --- | --- | --- | --- | --- | --- |
|  | **N** | **%** | **N** | **%** | **N** | **%** | **N** | **%** |  |
| ***Sociodemographic status, Ethnicity & conditions of living*** | | | | | | | | | |
| **Gender** | **172** |  | **85** |  | **126** |  | **383** |  | **0.90** |
| Male |  | 47.7% |  | 44.7% |  | 46.8% |  | 46.7% |  |
| Female |  | 52.3% |  | 55.3% |  | 53.2% |  | 53.3% |  |
| **Region** | **172** |  | **85** |  | **126** |  | **413** |  | **<0.001** |
| South |  | 49.4% |  | 56.5% |  | 73.8% |  | 60.1% |  |
| North |  | 32.6% |  | 40.0% |  | 19.8% |  | 28.3% |  |
| Islands |  | 18.0% |  | 3.5% |  | 6.4% |  | 11.6% |  |
| **Ethnicity** |  |  |  |  |  |  | **383** |  |  |
| Oceanian |  |  |  |  |  |  |  | 44.9% |  |
| European/ Others |  |  |  |  |  |  |  | 22.2% |  |
| Multiracial |  |  |  |  |  |  |  | 32.9% |  |
| **Place of living** | **165** |  | **84** |  | **123** |  | **372** |  | **<0.001** |
| Tribe/squat |  | 48.5% |  | 16.7% |  | 20.3% |  | 32.0% |  |
| Town/village/isolated property |  | 51.5% |  | 83.3% |  | 79.7% |  | 68.0% |  |
| **Health insurance** | **167** |  | **82** |  | **120** |  | **389** |  | **<0.001** |
| Basic public insurance only |  | 15.0% |  | 8.5% |  | 12.5% |  | 13.0% |  |
| State aid supplemental |  | 37.1% |  | 9.8% |  | 20.8% |  | 25.5% |  |
| Private supplemental |  | 47.9% |  | 81.7% |  | 66.7% |  | 61.5% |  |
| **Type of school** | **172** |  | **85** |  | **126** |  | **413** |  | **<0.001** |
| Public |  | 70.9% |  | 94.1% |  | 86.5% |  | 81.1% |  |
| Private |  | 29.1% |  | 5.9% |  | 13.5% |  | 18.9% |  |
| **Home sanitary equipment:** | **172** |  | **85** |  | **125** |  | **402** |  | **<0.001** |
| all available |  | 54.1% |  | 90.6% |  | 97.2% |  | 70.4% |  |
| one or more missing |  | 45.9% |  | 9.4% |  | 20.8% |  | 29.6% |  |
| ***Access to oral health care and prevention*** | | | | | | | | | |
| **number of sealed molars** | **172** |  | **85** |  | **126** |  | **413** |  | **0.001** |
| None |  | 48.8% |  | 34.1% |  | 27.8% |  | 38.7% |  |
| 1-3 |  | 35.5% |  | 35.3% |  | 43.6% |  | 38.1% |  |
| 4 |  | 15.7% |  | 30.6% |  | 28.6% |  | 23.2% |  |
| **Tooth brushing at school** | **172** |  | **85** |  | **125** |  | **412** |  | **0.55** |
| Yes |  | 48.8% |  | 45.9% |  | 42.4% |  | 46.1% |  |
| No |  | 51.2% |  | 54.1% |  | 57.6% |  | 53.9% |  |
| **Dental attendance** | **153** |  | **80** |  | **119** |  | **378** |  | **<0.001** |
| Never have visited |  | 20.3% |  | 11.25% |  | 14.3% |  | 16.2% |  |
| Already have visited a dentist |  | 79.7% |  | 88.75% |  | 85.7% |  | 83.8% |  |
| **Access to oral health care** | **172** |  | **85** |  | **126** |  | **403** |  |  |
| No difficulties |  | 44.8% |  | 77.65% |  | 53.2% |  | 54.8% | <0.001 |
| Perception of difficulties |  | 55.2% |  | 22.35% |  | 46.8% |  | 45.2% |  |
| **Participation to the OHP program** | **150** |  | **82** |  | **121** |  |  |  | **0.87** |
| Yes |  | 86.7% |  | 89.0% |  | 87.6% | 377 | 87.5% |  |
| No |  | 13.3% |  | 11.0% |  | 12.4% |  | 12.5% |  |
| ***Oral health behaviours*** | | | | | | | | | |
| **Frequency of tooth brushing** | **171** |  | **85** |  | **125** |  | **381** |  | **<0.001** |
| Twice a day or more |  | 43.9% |  | 76.5% |  | 60.8% |  | 56.7% |  |
| Once a day or less |  | 56.1% |  | 23.5% |  | 39.2% |  | 43.3% |  |
| **Usual drink when thirsty** | **172** |  | **85** |  | **125** |  | **382** |  | **0.85** |
| Sweet drink/Milk |  | 18.6% |  | 21.2% |  | 20.8% |  | 19.9% |  |
| Water |  | 81.4% |  | 78.8% |  | 79.2% |  | 80.1% |  |
| **Usual drink during mealtime** | **172** |  | **85** |  | **124** |  | **381** |  | **0.50** |
| Sweet drink/Milk |  | 34.3% |  | 27.1% |  | 32.3% |  | 32.0% |  |
| Water |  | 65.7% |  | 72.9% |  | 67.7% |  | 68.0% |  |
| **Sweet drinks during weekdays** | **172** |  | **85** |  | **126** |  | **383** |  | **0.004** |
| Never |  | 29.4% |  | 11.0% |  | 15.9% |  | 16.7% |  |
| Some days |  | 62.4% |  | 81.4% |  | 79.4% |  | 76.5% |  |
| Daily |  | 8.2% |  | 7.6% |  | 4.7% |  | 6.8% |  |
| **Sweet foods during weekdays** | **172** |  | **85** |  | **126** |  | **383** |  | **0.87** |
| Never |  | 8.7% |  | 15.3% |  | 9.5% |  | 10.4% |  |
| Some days |  | 86.6% |  | 80.0% |  | 88.9% |  | 85.9% |  |
| Daily |  | 4.65% |  | 4.7% |  | 1.6% |  | 3.7% |  |
| **Breakfast on weekdays** | **172** |  | **85** |  | **125** |  | **382** |  | **2.01** |
| No/ some days |  | 33.1% |  | 23.5% |  | 34.4% |  | 31.4% |  |
| Everyday |  | 66.9% |  | 76.5% |  | 65.6% |  | 68.6% |  |
