# Supplementary material for: Influence of an oral health promotion program on the evolution of dental status in New Caledonia: A focus on health inequities
Source: PLoS One. 2023 Oct 3;18(10):e0287067. doi: 10.1371/journal.pone.0287067 (PMC10547163; doi:10.1371/journal.pone.0287067)
Supplement: S3 Table — (DOCX) [file pone.0287067.s003.docx]

S3 Table: Comparison of caries indexes between 2012 and 2019 by province

|  | **South** | | **North** | | **Islands** | | **New Caledonia** | |
| --- | --- | --- | --- | --- | --- | --- | --- | --- |
|  | Mean (sd) | p* | Mean (sd) | p | Mean (sd) | p | Mean (sd) | p |
| **D_3_T** |  |  |  |  |  |  |  |  |
| 2012 | 0.58±1.12 |  | 0.35±0.72 |  | 0.57±1.00 |  | 0.53±1.04 |  |
| 2019 | 0.33±0.84 | 0.002 | 0.24±0.88 | 0.01 | 0.35±0.81 | 0.20 | 0.31±0.84 | <0.001 |
| **d_3_t** |  |  |  |  |  |  |  |  |
| 2012 | 1.40±1.91 |  | 1.72±2.01 |  | 2.29±2.03 |  | 1.56±1.96 |  |
| 2019 | 1.32±1.66 | 0.59 | 1.67±2.17 | 0.56 | 1.73±1.77 | 0.13 | 1.47±1.83 | 0.85 |
| **D_1_T** |  |  |  |  |  |  |  |  |
| 2012 | 1.10±1.48 |  | 1.41±1.38 |  | 1.7±1.47 |  | 1.23±1.47 |  |
| 2019 | 1.24±1.51 | 0.17 | 1.61±1.83 | 0.81 | 2.06±1.63 | 0.22 | 1.44±1.63 | 0.07 |
| **D_3_MFT** |  |  |  |  |  |  |  |  |
| 2012 | 0.82±1.38 |  | 0.59±1.01 |  | 0.66±1.16 |  | 0.76±1.29 |  |
| 2019 | 0.52±1.04 | <0.01 | 0.28±0.91 | <0.01 | 0.71±1.27 | 0.94 | 0.47±1.03 | <0.001 |
| **d_3_ft** |  |  |  |  |  |  |  |  |
| 2012 | 1.91±2.16 |  | 2.24±2.14 |  | .59±2.17 |  | 2.05±2.17 |  |
| 2019 | 1.92±1.20 | 0.78 | 2.07±2.24 | 0.47 | 2.58±2.06 | 0.33 | 2.04±2.08 | 0.91 |
| **D_1_MFT** |  |  |  |  |  |  |  |  |
| 2012 | 1.93±2.07 |  | 1.99±1.63 |  | 2.36±1.81 |  | 1.98±1.96 |  |
| 2019 | 1.77±1.97 | 0.61 | 1.90±1.97 | 0.38 | 2.77±1.53 | 0.34 | 1.92±1.95 | 0.10 |
| **d_3_ft+D_3_MFT** |  |  |  |  |  |  |  |  |
| 2012 | 2.74±2.87 |  | 2.83±2.48 |  | 3.25±2.66 |  | 2.81±2.7 |  |
| 2019 | 2.57±2.53 | 0.76 | 2.35±2.53 | 0.046 | 3.29±2.66 | 0.99 | 2.51±2.44 | 0.25 |
| **d_3_t+D_3_T index** |  |  |  |  |  |  |  |  |
| 2012 | 1.98±2.50 |  | 2.07±2.22 |  | 2.86±2.37 |  | 2.09±2.44 |  |
| 2019 | 1.66±1.94 | 0.77 | 1.91±2.46 | 0.22 | 2.08±2.22 | 0.05 | 1.78±2.13 | 0.17 |

*Mann-Whitney tests

2012: n=789, 2019: n=413
