# Supplementary material for: Influence of an oral health promotion program on the evolution of dental status in New Caledonia: A focus on health inequities
Source: PLoS One. 2023 Oct 3;18(10):e0287067. doi: 10.1371/journal.pone.0287067 (PMC10547163; doi:10.1371/journal.pone.0287067)
Supplement: S4 Table — (DOCX) [file pone.0287067.s004.docx]

S4 Table: Comparison of caries indexes between 2012 and 2019 by province - ZINB models

|  | **South** | | | | | **North** | | | | | **Islands** | | | | | **New Caledonia** | | | | |
| --- | --- | --- | --- | --- | --- | --- | --- | --- | --- | --- | --- | --- | --- | --- | --- | --- | --- | --- | --- | --- |
|  | mean±sd | Zero-inflated | | Negative binomial | | mean±sd | Zero-inflated | | Negative binomial | | mean±sd | Zero-inflated | | Negative binomial | | mean±sd | Zero-inflated | | Negative binomial | |
| Caries index |  | %d3t+D3t =0 N(%) | OR[IC] p* | d3t+D3t * value if d3t+D3t ≠0 | IRR[IC] p* |  | %d_3_t+D_3t_=0 N(%) | OR[IC] p* | d3t+D3t * value if d3t+D3t ≠0 | IRR[IC] p* |  | %d3t+D3t =0 N(%) | OR[IC] p* | d3t+D3t * value if d3t+D3t ≠0 | IRR[IC] P* |  | %d_3_t+D_3_t =0 N(%) | OR [IC] p* | d3t+D3t * value if d3t+D3t ≠0 | IRR[IC] P* |
| **D3T index** | | | | | | | | | | | | | | | | | | | | |
| 2012 | 0.58±1.12 | 381 (70.3%) |  | 1.94±1.26 |  | 0.35±0.72 | 128(76.6%) |  | 1.51±0.68 |  | 0.57±1.00 | 54 (67.5%) |  | 1.77±0.99 |  | 0.53±1.04 | 563(71.4%) |  | 1.85±1.15 |  |
| 2019 | 0.33±0.84 | 199 (80.2%) | 1.59 [0.81-3.12] 0.18 | 1.69±1.12 | 0.74[0.76-1.62] 0.20 | 0.24±0.88 | 104(88.9%) | 5[1.63-15.3] 0.004 | 2.15±1.72 | 2.11[0.99-4.44] 0.05 | 0.35±0.81 | 37 (77.1%) | 1.4[0.24-8.22] 0.71 | 01.54±1.03 | 0.72[0.26-2.00] 0.53 | 0.31±0.84 | 340(82.3%) | 2.11[1.22-3.65] 0.007 | 1.75±1.03 | 0.89[0.71-1.41] 0.54 |
| **d3t index** | | | | | | | | | | | | | | | | | | | | |
| 2012 | 1.40±1.91 | 277 (51.11%) |  | 2.87±1.81 |  | 1.72±2.01 | 71(42.51%) |  | 2.98±1.80 |  | 2.29±2.03 | 20 (25.0%) |  | 3.05±1.77 |  | 1.56±1.96 | 368(46.6%) |  | 2.92±1.80 |  |
| 2019 | 1.32±1.66 | 110 (55.6%) | 0.55 [0.33-0.90] 0.02 | 2.77±1.45 | 0.76[0.63-0.93] <0.001 | 1.67±2.17 | 54(46.1%) | 1.24[0.67-2.29] 0.48 | 3.11±2.06 | 1.05 [0.80-1.38] 0.7 | 1.73±1.77 | 18 (37.5%) | 1.89 [0.68-5.27] 0.22 | 2.77±1.45 | 0.88 [0.64-1.21] 0.45 | 1.47±1.83 | 182(44.1%) | 0.78[0.55-1.09] 0.15 | 2.63±1.72 | 0.86 [0.75-1.00] 0.04 |
| **D1T index** | | | | | | | | | | | | | | | | | | | | |
| 2012 | 1.10±1.48 | 276 (50.9%) |  | 2.24±1.38 |  | 1.41±1.38 | 61(36.5%) |  | 2.22±1.08 |  | 1.7±1.47 | 21 (26.2%) |  | 2.31±1.23 |  | 1.23±1.47 | 358(45.4%) |  | 2.25±1.23 |  |
| 2019 | 1.24±1.51 | 115 (46.4%) | 0.82 [0.52-1.29] 0.40 | 2.32±1.32 | 1.05[0.87-1.28] 0.61 | 1.61±1.83 | 51(43.6%) | 1.94[1.01-3.73] 0.04 | 2.86±1.53 | 1.42 [1.13-1.77] 0.002 | 2.06±1.63 | 12 (25.0%) | 1.35 [0.32-5.63] 0.68 | 2.75±1.27 | 1.27 [0.94-1.72] 0.118 | 1.44±1.63 | 178(43.1%) | 1.04[0.75-1.43] 0.81 | 2.54±1.39 | 1.19 [1.05-1.36] <0.01 |
| **D3MFT index** | | | | | | | | | | | | | | | | | | | | |
| 2012 | 0.82±1.38 | 339 (62.5%) |  | 2.20±1.44 |  | 0.59±1.01 | 115(68.9%) |  | 1.88±0.92 |  | 0.66±1.16 | 52 (65.0%) |  | 1.89±1.23 |  | 0.76±1.29 | 506(64.1%) |  | 2.11±1.35 |  |
| 2019 | 0.52±1.04 | 179 (72.2) | 1.38 [0.79-2.42] 0.26 | 1.87±1.16 | 0.74[0.52-1.04] 0.08 | 0.28±0.91 | 101(86.3%) | 3.48[1.64-7.38] 0.001 | 2.06±1.57 | 1.39 [0.68-2.00] 0.56 | 0.71±1.27 | 32 (66.7%) | 1.48 [0.28-7.86] 0.64 | 2.12±1.36 | 1.25 [0.56-2.78]0.58 | 0.47±1.03 | 312(75.5%) | 1.78[1.18-2.70] <0.01 | 1.94±1.25 | 0.86 [0.65-1.13] 0.27 |
| **D1MFT index** | | | | | | | | | | | | | | | | | | | | |
| 2012 | 1.93±2.07 | 205 (37.8%) |  | 3.10±1.81 |  | 1.99±1.63 | 42(25.1%) |  | 2.66±1.33 |  | 2.36±1.81 | 16 (20.0%) |  | 2.95±1.54 |  | 1.98±1.96 | 263(33.3%) |  | 2.98±1.68 |  |
| 2019 | 1.77±1.97 | 97 (39.1%) | 1.00 [0.68-1.48] 0.96 | 2.90±1.77 | 0.92[0.79-1.06] 0.26 | 1.90±1.97 | 46(39.3%) | 0.21[0.13-0.36] <0.01 | 3.13±1.60 | 1.22 [1.01-1.47] 0.04 | 2.77±1.53 | 6 (12.5%) | 0.50 [0.10-2.48] 0.4 | 3.17±1.19 | 1.09 [0.85-1.88]0.50 | 1.92±1.95 | 149(36.1%) | 1.16[0.86-1.57] 0.33 | 3.00±1.64 | 1.01 [0.91-1.12] 0.83 |
| **d3ft+D3MFT index** | | | | | | | | | | | | | | | | | | | | |
| 2012 | 2.74±2.87 | 172 (31.7%) |  | 4.01±2.63 |  | 2.83±2.48 | 36(21.6%) |  | 3.61±2.24 |  | 3.25±2.66 | 14 (17.5%) |  | 3.94±2.42 |  | 2.81±2.7 | 222(28.1%) |  | 3.91±2.42 |  |
| 2019 | 2.44±2.34 | 63 (25.4%) | 0.48 [0.26-0.90] 0.02 | 3.27±2.14 | 0.78[0.67-0.90] <0.001 | 2.35±2.53 | 39(33.3%) | 2.26[1.00-5.10] 0.049 | 3.52±2.33 | 0.97 [0.77-1.21] 0.80 | 3.29±2.66 | 9 (18.75%) | 1.16 [0.32-4.17] 0.82 | 4.05±2.37 | 1.03 [0.78-1.36] 0.82 | 2.51±2.44 | 111(26.9%) | 0.79[0.52-1.19] 0.253 | 3.44±2.23 | 0.85 [0.76-0.96] 0.007 |
| **d3t+D3T index** | | | | | | | | | | | | | | | | | | | | |
| 2012 | 1.98±2.50 | 242 (44.65%) |  | 3.57±2.36 (n=300) |  | 2.07±2.22 | 58(34.7%) |  | 3.17±2.01 (n=109) |  | 2.86±2.37 | 16 (20.0%) |  | 3.58±2.11 (n=64) |  | 2.09±2.44 | 316(40.0%) |  | 3.48±2.25 (n=473) |  |
| 2019 | 1.66±1.94 | 154 (62.1%) | 0.48 [0.27-0.83] <0.01 | 2.67±1.84 (n=154) | 0.67[0.56-0.81] <0.001 | 1.91±2.46 | 66(56.4%) | 1.77[0.88-3.57] 0.11 | 3.39±2.38 (n=66) | 1.09 [0.83-1.43] 0.53 | 2.08±2.22 | 32 (66.7%) | 2.20 [0.67-7.18] 0.19 | 3.12±2.03 (n=32) | 0.84 [0.61-1.17] 0.31 | 1.78±2.13 | 161(39.0%) | 0.77[0.53-1.12] 0.17 | 2.92±2.03 (n=252) | 0.79[0.69-0.91] <0.001 |

Zero-Inflated Negative Binomial models without random effects, 2012: n=789, 2019: n=413
