# Supplementary material for: Influence of an oral health promotion program on the evolution of dental status in New Caledonia: A focus on health inequities
Source: PLoS One. 2023 Oct 3;18(10):e0287067. doi: 10.1371/journal.pone.0287067 (PMC10547163; doi:10.1371/journal.pone.0287067)
Supplement: S5 Table — (DOCX) [file pone.0287067.s005.docx]

S5 Table: Bivariate analysis: Relationship between caries experience and explanatory variables **(2019)**

| Variable name | **n** | **d_3_t+D_3_t *** | **Zero-inflated negative binomial regression** | | | |
| --- | --- | --- | --- | --- | --- | --- |
|  |  |  | **% d_3_t+D_3_t =0**  **N (%)** | **OR [95% CI] p** | **Mean d_3_t+D_3_t if ≠0** | **IRR[95% CI] p** |
| **Gender** | *413* |  |  |  |  |  |
| Male |  | 2.02±2.2 (n=196) | 68 (34.7%) |  | 3.09±2 (n=128) |  |
| Female |  | 1.56±2.06 (n=217) | 93 (42.9%) | 1.4 [0.62-3.17] p=0.42 | 2.73±2.06 (n=124) | 0.83 [0.65-1.07] p=0.16 |
| **Region** | *413* |  |  |  |  |  |
| South |  | 1.65±1.94 (n=248) | 94 (37.9%) |  | 2.67±1.84 (n=154) |  |
| North |  | 1.91±2.46 (n=117) | 51 (43.6%) | 1.97 [0.64-6.05] p=0.24 | 3.39±2.38 (n=66) | 1.44 [1.05-1.98] p=0.02 |
| Islands |  | 2.08±2.22 (n=48) | 16 (33.3%) | 1.09 [0.23-5.19] p=0.91 | 3.12±2.03 (n=32) | 1.27 [0.84-1.92] p=0.25 |
| **Ethnicity** | *383* |  |  |  |  |  |
| European/ Others |  | 0.85±1.52 (n=85) | 47 (55.3%) |  | 1.92±1.78 (n=38) |  |
| Oceanian |  | 2.31±2.3 (n=172) | 50 (29.1%) | 0.45 [0.1-1.95] p=0.29 | 3.26±2.1 (n=122) | 2.28 [1.48-3.53] p<0.001 |
| multiracial |  | 1.48±1.95 (n=126) | 56 (44.4%) | 1.17 [0.28-4.94] p=0.83 | 2.66±1.92 (n=70) | 1.77 [1.11-2.82] p=0.02 |
| **Place of living** | *395* |  |  |  |  |  |
| Town/village/isolated property |  | 1.57±1.97 (n=267) | 113 (42.3%) |  | 2.72±1.9 (n=154) |  |
| Tribe/squat |  | 2.15±2.34 (n=150) | 42 (32.8%) | 0.6 [0.2-1.84] p=0.38 | 3.2±2.19 (n=86) | 1.25 [0.96-1.64]  p=0.1 |
| **Health insurance** | *389* |  |  |  |  |  |
| Private supplemental |  | 1.45±1.98 (n=239) | 112 (46.9%) |  | 2.72±1.97 (n=127) |  |
| State aid supplemental or Basic insurance |  | 2.28±2.24 (n=98) | 38 (25.33%) | 0.10 [0.001- 6] p=0.28 | 3.05±2.09 (n=112) | 1.18 [0.90-1.55] p=0.23 |
| **Type of school** | *413* |  |  |  |  |  |
| Public |  | 1.72±2.16 (n=335) | 138 (41.2%) |  | 2.93±2.1 (n=197) |  |
| Private |  | 2.01±2 (n=78) | 23 (29.5%) | 0.36 [0.05-2.5] p=0.3 | 2.85±1.79 (n=55) | 0.97 [0.68-1.37] p=0.85 |
| **sanitary equipment** | 402 |  |  |  |  |  |
| has all the equipment |  | 1.5±1.94 (n=282) | 126 (44.7%) |  | 2.7±1.89 (n=156) |  |
| one or more missing equipment |  | 2.4±2.39 (n=120) | 31 (25.8%) | 0.25 [0.04-1.5] p=0.13 | 3.24±2.24 (n=89) | 1.23 [0.99-1.68] p=0.06 |
| **number of sealed molars** | *413* |  |  |  |  |  |
| =4 |  | 1.08±1.41 (n=91) | 43 (47.25%) |  | 2.04±1.34 (n=48) |  |
| <4 |  | 1.79±2.12 (n=158) | 57 (36.1%) | 1.2 [0.23-6.3] p=0.83 | 2.8±2.05 (n=101) | 1.7 [1.16-2.49]  p<0.01 |
| None |  | 2.16±2.38 (n=164) | 61 (37.2%) | 1.62 [0.33-7.84] p=0.55 | 3.44±2.14 (n=103) | 2.24 [1.53-3.26] p<0.001 |
| **Participation to the OHP program** | 377 |  |  |  |  |  |
| Yes |  | 1.65±2.09 (n=328) | 137 (41.8%) |  | 2.84±2.03 (n=191) |  |
| No |  | 2±2.2 (n=49) | 17 (34.7%) | 0.89 [0.24-3.31] p=0.86 | 3.06±2.03 (n=32) | 1.1 [0.74-1.61]  p=0.66 |
| **Tooth brushing at school** | *412* |  |  |  |  |  |
| No |  | 1.66±2.06 (n=219) | 87 (39.7%) |  | 2.75±2.01 (n=132) |  |
| Yes |  | 1.91±2.21 (n=193) | 74 (38.3%) | 1.02 [0.4-2.56] p=0.97 | 3.1±2.06 (n=119) | 01.18 [0.9-1.55] p=0.23 |
| **Dental attendance** | *378* |  |  |  |  |  |
| Had visited a dentist |  | 1.77±2.11 (n=318) | 129 (40.6%) |  | 2.97±1.98 (n=189) |  |
| Had never visited a dentist |  | 1.91±2.14 (n=60) | 17 (28.3%) | 0.3 [0.04-2.1] p=0.23 | 2.67±2.09 (n=43) | 0.88 [0.63-1.24] p=0.47 |
| **Access to oral health care** | *403* |  |  |  |  |  |
| No difficulties |  | 1.16±1.65 (n=218) | 115 (52.75%) |  | 2.46±1.6 (n=103) |  |
| Difficulties |  | 2.48±2.38 (n=185) | 42 (22.7%) | 0.14 [0.03-0.62] p<0.01 | 3.2±2.24 (n=143) | 1.47 [1.13-1.91] p<0.01 |
| **Frequency of tooth brushing** | *411* |  |  |  |  |  |
| Twice a day; |  | 1.38±1.87 (n=232) | 108 (46.5%) |  | 2.59±1.86 (n=124) |  |
| Less than twice a day |  | 2.28±2.35 (n=179) | 53 (29.6%) | 0.38 [0.13-1.11] p=0.08 | 4.68±3.16 (n=126) | 1.34 [1.03-1.74] p=0.03 |
| **Usual drink when thirsty** | *412* |  |  |  |  |  |
| Water |  | 1.71±2.12 (n=327) | 135 (41.3%) |  | 2.92±2.03 (n=192) |  |
| Sweet drink/Milk |  | 2.02±2.19 (n=85) | 26 (30.6%) | 0.45 [0.11-1.82] p=0.26 | 2.91±2.08 (n=59) | 1.04 [0.76-1.41] p=0.81 |
| **Usual drink during mealtime** | *411* |  |  |  |  |  |
| Water |  | 1.61±2.03 (n=277) | 120 (43.3%) |  | 2.84±1.95 (n=157) |  |
| Sweet drink/Milk |  | 2.13±2.3 (n=134) | 41 (30.6%) | 0.4 [0.12-1.37] p=0.15 | 3.06±2.18 (n=93) | 1.1 [0.84-1.44]  p=0.47 |
| **Frequency of consumption of sweet drinks during weekdays** | *403* |  |  |  |  |  |
| No day |  | 1.1±1.74 (n=67) | 36 (53.7%) |  | 2.39±1.87 (n=31) |  |
| Some days/ Everyday |  | 1.9±2.16 (n=336) | 121 (36%) | 0.32 [0.07-1.46] p=0.14 | 2.97±2.04 (n=215) | 1.37 [0.89-2.1]  p=0.15 |
| **Frequency of consumption of sweet foods during weekdays** | *403* |  |  |  |  |  |
| No day |  | 1.05±1.67 (n=42) | 19 (45.2%) |  | 1.91±1.85 (n=23) |  |
| Some days /Every day/ |  | 1.85±2.15 (n=361) | 138 (38.2%) | 2.4 [0.13-41] p=0.55 | 2.99±2.02 (n=223) | 2.07 [1.25-3.43] p<0.01 |
| **Frequency of taking breakfast on weekdays before school** | *402* |  |  |  |  |  |
| Every day/daily |  | 1.55±2.03 (n=276) | 123 (44.6.9%) |  | 2.8±1.99 (n=153) |  |
| No day/ Some days |  | 2.21±2.23 (n=126) | 34 (27%) | 0.21 [0.03-1.29] p=0.09 | 3.02±2.09 (n=92) | 1.16 [0.88-1.54] p=0.29 |

* Mean± standard deviation, OR = odds ratio, IRR: incidence rate ratio, 95%CI: 95% Confidence Interval

+ Zero-inflated negative binomial regression with random effects
