# Supplementary material for: Influence of an oral health promotion program on the evolution of dental status in New Caledonia: A focus on health inequities
Source: PLoS One. 2023 Oct 3;18(10):e0287067. doi: 10.1371/journal.pone.0287067 (PMC10547163; doi:10.1371/journal.pone.0287067)
Supplement: Attachment — Submitted filename: Response to Reviewers.docx [file pone.0287067.s006.docx]

Reviewer's Responses to Questions

Reviewer #1: In materials and methods, it should include the inclusion and exclusion criteria of the study sample.

The study population was made of all schoolchildren aged 9, in New Caledonia in 2019-2020 (NB school is compulsory in NC, Almost 100% of 9-years old children are going to school). They thus all had a non-null and known probability of being selected for being part of the study sample. This is a random sampling process, as usual in descriptive epidemiological surveys.

The following sentence has been modified as follow to make it clearer : " The study population **(All 9 years old schoolchildren in NC in 2019-2020)** was stratified according to the province (Islands, North, South), the school area (Islands: Lifou, Maré, Ouvéa; North: Grand Nord, North-East, North-West; South: South-East, South-West) and the type of school (public, private).

In the data collection section of the study variable, could you clarify what were the criteria and/or indicator and the procedure used to record sealants in permanent molars?

Dentists have checked the presence of dental sealants (visually + with the use of a dental probe) for each permanent molar on the occlusal surface and indicated if there was no sealant (no sign of the presence of dental sealant material), partial sealing of the occlusal fissures (some part of the occlusal fissures with dental sealant material, effective sealing not ensured) or complete presence of the dental sealant (all occlusal fissures with sealant material, sealing ensured) . Some more information have been included table S1 to make it clearer.

**“+ no sign of dental sealant material vs partial (some part of the occlusal fissures with dental sealant material) or complete (all occlusal fissures with sealant material) presence of dental sealant”**

In the discussion, the importance of the study should be argued, that is, why it is important to measure the impact of health promotion interventions on oral health considering health inequities

A sentence has been added in the discussion to emphasize this aspect **“Thus, measuring the impact of health promotion interventions on oral health considering health inequities is very important. Indeed, OHP intervention can be effective for the whole population with nonetheless an increase in health inequities if the positive effects are lowered in children with higher health need.**

Reviewer #2:

Line 1. The impact evaluation implies having a real comparison group or a counterfactual and a clear health outcome in which they will evaluate the "impact", for how the article is I suggest to review it carefully.

The authors agree that the term “impact” might be inappropriate in view of the study methodology. We have thus replaced the term “impact of the program” by words such as “**role/influence of the program on the evolution of dental status**”. The title also has been changed and is now : **Influence of an oral health promotion program on the evolution of dental status in New Caledonia : a focus on health inequities.**

Line 36. Review the use of abbreviations throughout the manuscript for consistency.

We have reviewed the abbreviations in the text to make them more consistent

Line 89. Why only 9 years old group?

In 2012, the ages of 6,9 and 12 were chosen as they represent key ages for epidemiological studies on dental status. The program started in 2014 and is mainly focused on schoolchildren (age 5 and more). Thus 9 years old children in 2019 had benefited from the program since start (4 years old in 2014). The analyses are complex with many tables. It was not possible to give data for all the age groups in one manuscript. We think of submitting later other manuscripts for 6 and 12 years old children, with different insights. The sentence has been modified to make it clearer “ This manuscript focuses on 9-years-old children, **who benefited from the program since start,** with the objective of identifying the determinants and risk factors explaining the presence of untreated dental caries in 2019”

Line 98. It is not clear the level of representativeness, is it national? only for NC? or is it part of a national survey where one of the provinces where data was collected is NC?

The study concerns New Caledonia at a whole and the sample is representative of the population of the NC country. NC can be considered as a "country" due to its large autonomy but at the same time it is a French territory. We have thus deleted the term "national" to avoid confusion and have changed the word “country” by the word “**territory**”.

Line 104-108. How much was the prevalence and power? Did you compute the ICC? If so, how much is it?

It was an accuracy calculation so power was not used in the calculation formula

Prevalence was set at 40%: this is now indicated in the text.

The ICC was verified in the 2019 database and was equivalent to the 2012 one (approx. 0,05)

Line 109. Which rules-of-thumbs?

There is no clear rule as recommendations vary from 15 to 30 subjects per variable depending on the authors. In order to avoid confusion we have changed the word rules-of-thumbs and replaced it by the term “recommendation”

Line 105 vs line 111. It's a little confusing that they put the required sample size and then the sufficient sample size.

We are not sure to have understood this comment but to avoid confusion the word “ estimated” is now used instead of “sufficient or required”

Line 129. You do not say before what ICDAS is

The acronym is now defined in the text

Line 134. And not the adult responsible for the child) (who are not necessarily the parents)

This has been added in the text

Line 136. Province of residence or birth?

This is now clarified (province of residence)

Line 149. What kind of errors or biases?

A sentence has been added : “**Differences observed in caries detection were concerning mainly inter-examiner variations for initial stages (1 to 3), they did not change much caries indexes calculations**”.

Line 153. You did not previously describe the severity

The definition is now given as follow : “A descriptive analysis was conducted to describe the prevalence **(% of children with disease)** and severity of dental diseases **(mean caries indexes)** and present the characteristics of the study population”.

Line 160. “Complete” means all age groups?

It means initial/original, complete (no missing data), raw datasets. The wording has been changed to “**initial raw**”

Line 162. ICC was considered in the models?

The ICC was not considered for the initial descriptive and exploratory analyses. But, mixed models were used that integrate the school and examiner effects in the models

FYI : we have checked the ICC for the investigator effect, Interobserver ICC(A,1) = 0.896 (0.827 < ICC < 0.945), Intra-Observer ICC mean = 0,9406 (data not shown)

Line 187. Did you consider using interaction terms or focusing the variables?

Interactions between covariates were explored when building the final model.

Significant interactions were found between

- ethnicity and the number of sealed molars, difficulties in accessing oral healthcare, consumption of sugary food, frequency of toothbrushing

- the number of molar sealed and difficulties in accessing oral healthcare, frequency of toothbrushing ,

- difficulties in accessing oral healthcare and consumption of sugary food, frequency of toothbrushing,

Interactions were multiple and not clinically obviously interesting.

We felt that their integration in the model would have complexified it much. Thus we chose not to saturate the model and to focus on the proper effect of the explanatory variables. Namely, we considered that there was a possible lack of power to interpret these interactions

A sentence has been added in the text to make this clearer.

Line 202. Table 1. Use Total instead of “NC”, or the complete name.

Total is now used in table 1 ( and others)

• Health insurance: What type of services or care the first two categories cover.

Information has been added table S1 to help the reader understand what is covered

***Basic public health insurance in NC is covering a large proportion of essential oral health care fees. Supplementary state aid (for low income earners) and private insurances complete this dental coverage and potentially give extra reimbursements for other types of dental treatments such as prosthetic treatments. Dental care is delivered in private practices or in public structures.**

• Dental attendance: How many is “high”?

The term high has been changed to « **Visit to the dentist every year**” in Table 1 and S1

• Access to oral health: What mean “no difficulties”

It is now indicated Table S 1 that it relates to the question **“ During the last year, did you attempt to attend the dentist but could not get an access to oral health care for your child?**

Line 221. Are you sure that mean and SD are the best summary statistics? (I guess median and IQR) please, evaluate the distribution.

The authors have chosen to present mean and SD in this descriptive table as it is an usual way of presenting data about dental status worldwide. The objective is to facilitate international comparisons. Moreover, the variables were presented according to their statistical distribution. For example for D_3_MFT, mean & SD are given along with the number of children with at least one tooth affectedThe skewed distribution of the indexes has been taken into account in the multidimensional explanatory analyses namely with the ZINB model.

Line 224. Table 2 I strongly suggest not to use abbreviations for variables, better to put them at the foot of the table.

A legend has been added at the foot of the table

***Mean and SD, and percentages with 95% confidence interval**

**DMFT : Number of Decayed, Missing and Filled Teeth for permanent teeth**

**dft : Number of Decayed and Filled Teeth for temporary teeth**

**caries detection threshold D1 : ICDAS stages 1 and more, D3 : ICDAS ICDAS stages 4 and more**

Line 250. Why did you fraction the models?

We had a high number of explanatory variables (lots of multi-colinearity) and we have considered that it was pertinent to progress step by step by following a conceptual framework with 3 models : social determinants, risk factors and acesss to oral health care and prevention

Line 320. Is a strong limitation for the comparison of the two years, I suggest explaining it better.

The changes in caries detection criteria, as explained in the discussion, may have jeopardised the comparisons for dental indexes that integrated initial lesions. For later carious stages (D3/ ICDAS 4 and more), there were not much differences in the criteria used between 2012 and 2019. This is the reason why the comparisons are focusing on D3 indexes. Moreover what is interesting in this study is the variations in the evolution between provinces that is not much influenced by the changes in the criteria. One sentence has been added in the material and method section to clarify that aspect (cf previous comment) as well as another one in the discussion : “**However, there were not much differences between 2012 and 2019 in the criteria used for the detection of more advanced lesions , which facilitated the comparisons for indexes based on the D3 threshold**.”

Would expect more discussion on the impact of oral health programs in the province, country or region.

The discussion has been modified slightly in view of this comment as we don’t know much yet about the differences in the implementation of the program depending of the provinces. this is being evaluated.

NB The concepts of country (NC territory) , province ( 3 differents parts of NC: north, south, islands) and region ( pacific region) have been harmonized in the text

Review whether they are actually doing an evaluation or measurement of impact.

The study is (partly) relating to a measurement of impact but the program is being evaluated by the health agency so that to identify its strengts, limits or the variations in the conditions of implementation. The results of the program evaluation are not fully available yet due to the delays due to the COVID 19 pandemic. This is now better explained in the discussion.

Suggest putting in context how the province is doing relative to the country, suggest using disease burden articles:

Results have been put in context in the discussion using the recent WHO oral health report
